# The impact of intraoperative optical coherence tomography on cognitive load in virtual reality vitreoretinal surgery training

**DOI:** 10.1038/s41598-025-07670-7

**Published:** 2025-07-10

**Authors:** Laura Schütz, Shervin Dehghani, Michael Sommersperger, Koorosh Faridpooya, Nassir Navab

**Affiliations:** 1 Computer Aided Medical Procedures and Augmented Reality, Technical University of Munich, Munich, Germany; 2 Rotterdam Eye Hospital, Rotterdam, The Netherlands

**Keywords:** Cognitive load, Optical coherence tomography, Ophthalmic surgery, Physiological computing, Image-guided surgery, Virtual reality, Medical imaging, Surgery, Biomedical engineering, Working memory

## Abstract

Ophthalmic surgeries consist of highly demanding microsurgical tasks that require surgeons to sustain mental focus and extreme manual dexterity for extended periods. To improve surgical precision and decision-making, intraoperative Optical Coherence Tomography (iOCT) has been integrated into surgical microscopes, offering cross-sectional imaging of anatomical tissues and instruments alongside the traditional microscopic view. While the clinical benefits of iOCT have been demonstrated across various ophthalmic procedures, the impact of this auxiliary information on the surgeon’s cognitive load has not yet been explored. This work is the first study to investigate physiological and subjective cognitive load during simulated iOCT-guided retinal surgery in 17 novice users. The eye tracking capabilities of a virtual reality eye surgery simulator were used to capture blink rate and pupil diameter. Electrocardiography was used to record heart rate (HR) and heart rate variability (HRV). iOCT-guided surgery resulted in significantly higher cognitive load than traditional microscope-guided surgery, indicated by increased HR (p<0.01), HRV (p<0.05), subjective task load (p<0.001) (NASA-TLX, mental effort), and task time (p<0.001). However, our findings indicate that although iOCT initially imposed a higher cognitive load on novice users, it ultimately enhanced their depth-related targeting precision, which is crucial for improving surgical accuracy and overall outcomes in vitreoretinal surgery.

## Introduction

Vitreoretinal surgeries are complex procedures that require extreme manual dexterity. During such interventions, microsurgical instruments are introduced to the backside of the eye to delicately manipulate small tissue structures of the on average $250 \mu m$ thin^1^ retina. For instance, during intra- or sub-retinal injection, a micron-cannula needs to be precisely inserted into the retinal tissue to inject a therapeutic agent. Another example is retinal membrane peeling, where a membrane as thin as $60 \mu m$^2^ is removed from the retinal surface. As a manual tremor of $182 \mu m$ RMS^3^ can be measured for ophthalmic surgeons, such tasks naturally pose a high physical and mental demand on surgeons. This is further exacerbated as the primary visual access to the surgical site is provided by an operating microscope, exclusively allowing a top-down perspective. This combination of limited viewing access and a physically and mentally demanding task requires years of training and experience before surgeons reach a sufficiently high and stable success rate for complex retinal procedures^4^.

In modern surgical systems, microscope-integrated intraoperative Optical Coherence Tomography (iOCT) provides additional imaging. iOCT can generate depth-resolved 2D cross-sectional images, referred to as B-scans, of the retina that reveal fine retinal layers and delicate tool-tissue interactions at a micrometer resolution. Leveraging this advanced technology, surgeons benefit from intraoperative visualization of small tissue structures and positional information regarding surgical instruments. The clinical benefit of iOCT has been demonstrated in several studies, highlighting its role in enhancing intraoperative decision-making^5^. It has been proven valuable in providing insights into tissue configurations during surgery, allowing for precise assessment of retinal conditions^6^. In particular, for retinal membrane peeling, it has been well-documented that iOCT can confirm successful membrane peeling without residual membranes, reducing the likelihood of reoperation^7^. Furthermore, iOCT guidance can enable precise instrument navigation, such as during needle insertion for subretinal injection^8–10^.

However, a current shortcoming of iOCT guidance in available surgical systems is the display of iOCT images to the surgeon with a spatial offset next to the microscope view. This circumstance requires surgeons to perform mental mapping when relating the spatial location of the structures apparent in the B-scans (iOCT images) to their corresponding location in the microscope view. Instrument navigation based on iOCT images is additionally challenging, as iOCT-typical imaging artifacts complicate image interpretation. The most prominent artifacts are instrument-induced shadows that obscure underlying retinal structures, as well as mirroring artifacts causing structures that are spatially located above the imaging area to appear mirrored within the iOCT image^11^. Furthermore, iOCT use has been reported to prolong surgery duration^6^. Hence, although iOCT can enable improved surgery outcomes, an adequate level of familiarity with the imaging modality is imperative for efficient surgical utility.

To make the additional anatomical information from iOCT images more accessible for surgeons, advanced visualization techniques, data fusion, multisensory feedback, or AI-guided user interfaces (UIs) are required. In this effort to improve the usability and interpretability of iOCT images, several works have proposed advanced visualization^12–16^ or sonification methods^17,18^. While these works are focused on evaluating alternative ways to display iOCT data, the effects of utilizing iOCT B-scans with currently employed user interfaces on the user’s cognitive load remained unexplored. We hypothesize that the presence of these additional visual stimuli (iOCT B-scans) side by side the standard microscope view leads to increased cognitive load, especially among novice users. With the ultimate goal of providing surgeons with the maximum amount of information possible while effectively managing their cognitive resources, it becomes increasingly important to develop and validate methods for measuring surgeons’ cognitive load.

Therefore, in this paper, we investigated the impact of iOCT, on cognitive load during vitreoretinal surgery. To our knowledge, this is the first work to measure and evaluate the cognitive load during ophthalmic surgery when using iOCT information in addition to the traditional microscopic view. We used an existing Virtual Reality (VR) eye surgery simulation system and asked 17 novice users to perform a spatial targeting task in simulated microscopic- and iOCT-guided surgery. During the study, we collected physiological and subjective measures of cognitive load. Physiological responses were captured using the head-mounted display’s (HMD) integrated eye tracking. An electrocardiogram (ECG) was used to capture cardiac indicators.

Our work makes the following main contributions:

- Assessment of cognitive load during simulated vitreoretinal surgery.
- Report of physiological and subjective cognitive load during iOCT-guided and microscope-guided vitreoretinal surgery in 17 novice users.
- Insights into the learnability of iOCT: novice users achieved greater depth-related targeting precision in iOCT-guided surgery compared to microscope-guided surgery.

## Related work

### Cognitive load theory

Cognitive load indicates how much of a learner’s finite working memory capacity is engaged during a learning activity^19^. Cognitive load theory provides a distinction into intrinsic, extraneous, and germane cognitive load. While intrinsic cognitive load stems from the difficulty of a task itself, extraneous cognitive load is imposed by the means in which information is presented. Germane cognitive load is associated with processing information and creating schemas during learning activities^19^. Intrinsic cognitive load is the most relevant to tasks in eye surgery as it relates to the interactivity of elements. In surgical tasks multiple interactive elements are present at the same time. During eye surgery, for example, the following interactive elements might be present: light probe, surgical instrument along with its shadow cast on the retina, anatomical target, and two iOCT images (B-scans). For this exemplary visuospatial motoric task, the manipulation and processing of the mentioned elements need to be learned at the same time as their interaction is critical to the task. For instance, only learning how to manipulate the light probe will not suffice to successfully execute the task. The light probe, instrument, and iOCT images are concurrent interactive parts of the task that are present during learning efforts. In these cases where interactions between elements need to be learned, intrinsic cognitive load will be high^20^. It is known that human working memory has limited capacity. Only about 7 items can be retained in the working memory processor at a time^21^. For interactive tasks, the number of elements that can be processed simultaneously is reduced to two or three^22^, making eye surgery tasks highly demanding.

It is important to note that cognitive load per se is an inherent part of learning efforts. However, the instructional design has to take into consideration limited human processing capacity, especially when faced with a new task^20^. Adapting task difficulty to the learner’s cognitive load is desirable to avoid situations of both too much and too little load to keep the learner engaged and motivated. An exemplary system for piano learning adapting to the user’s cognitive load has been shown to result in faster and better skill acquisition^23^.

### Cognitive load measurements

Cognitive load measurements can be categorized into subjective, performance-based, and physiological measurement techniques^24^. Exemplary subjective measurement techniques are the Subjective Workload Assessment Technique (SWAT)^25^ and the NASA Task Load Index (NASA-TLX)^26^. Performance-based measurement techniques refer to the primary task performance measured in task time and accuracy. The speed and accuracy of primary task execution will drop with increasing mental workload. Among physiological measurements of cognitive load are heart rate, brain activity, and eye activity^24^. In this work, we employed multimodal cognitive load measurements from all three categories. A raw NASA-TLX served as a subjective assessment, task time and targeting precision as performance-based metrics, and ocular and cardiac measures as physiological indicators of cognitive load.

#### Physiological cognitive load measurements

Heart rate (HR) and heart rate variability (HRV) are proven to be reliable indicators of cognitive load^27,28^ as they are a function of psychological changes and can indicate task difficulty^29^. While heart rate denotes the number of heartbeats occurring within a minute, heart rate variability reflects the variability in the time intervals between successive heartbeats. HR and HRV can be measured using an ECG. The interval between two consecutive R waves (peaks visible in an ECG signal), also referred to as RR, is the basis of many HRV measures^30^. While heart rate is easy to measure and can be viewed as an indicator of mental effort, it is easily influenced by diverse psychological and physical circumstances^29^. HRV, however, has shown to be a reliable measure of stress^31^ and cognitive load^32^, especially for short-term tasks^33^. HR and HRV have also been used as indirect markers of surgeons’ intraoperative stress^34^.

Eye metrics are another source of strong psychophysiological indices of cognitive load. Pupil diameter (PD), blink rate (BR), and gaze fixation duration are exemplary eye indices. PD is influenced by the level of cognitive activity^35^. A larger PD, measured in millimeters (mm), indicates higher problem difficulty^36^. During short-term memory tasks, PD can give insight into the amount of material that is actively processed at a time. Therefore, the rate of change in pupil diameter is a function of task difficulty^37^. To capture pupil activity in a way that relates to cognitive load, Duchowski et al. have introduced the Index of Pupillary Activity (IPA), a measure of the frequency of pupil diameter oscillations. A higher IPA indicates a greater task difficulty^36^. Blink rate has been reported as another physiological indicator of cognitive load. While a human will, on average, blink 23.4 times per minute during a resting state^38^, the blink frequency will increase during moments of heightened workload^39^ and decrease during tasks that require intensive visual processing (e.g., during reading or air traffic control)^40^. Gaze fixation duration is yet another ocular indicator of cognitive load. Longer fixation periods have been stated to refer to increased cognitive load^41^.

#### Physiological cognitive load in healthcare

Physiological measures promise to be an objective method of assessing cognitive load. Particularly in medical applications, were users are engaged in complex medical procedures of a few minutes up to a few hours, continuous assessment is of great interest. Therefore, physiological measures have found application in the medical domain, e.g. for assessing clinical decision-making^28^, surgical skill^42–44^, or cognitive load during surgery^34,45,46^. Many of those studies record physiological measures along with traditional subjective measures looking for correlations between the two categories to prove the validity of physiological parameters as a substitution or addition to subjective cognitive load evaluation^44,46,47^.

One study that evaluated the effect of cognitive load on clinical decision-making showed that cognitive load and HRV are indirectly correlated. Increasing cognitive load led to decreasing HRV^28^.

Researchers have also been interested in assessing physicians’ skill levels using physiological cognitive load measures. One of these studies recorded HRV of two cardiac surgeons during fifty coronary artery bypass grafting surgeries. A clear difference in HRV between the attending and consultant surgeon was found^42^. Gunawardena et al. reported that pupil diameter and pupillary index are reliable metrics in differentiating skill levels during laparoscopic surgery^43^. Another work involving twenty-nine medical trainees and clinicians predicted clinical ultrasound acquisition performance from cognitive load. They established an association between the gaze shift rate and a subjective measure of cognitive load within a clinical training setting. The study furthermore reported that novices showed poorer performance when experiencing high cognitive load, whereas experienced physicians managed to sustain good performance levels despite increased cognitive demand^44^.

Lastly, there are a number of works reporting on physiological measures of cognitive load during surgery. One study reported that the mental strain measured via heart rate variability varied between laparoscopic and open sigmoid resections^45^. Another study by Johannessen et al.^47^ captured pupil size, galvanic skin response, and heart rate of five trauma physicians during real-life resuscitations. They reported the strongest correlation of the skin conductance response amplitude and frequency as well as the eye tracking metrics with subjective mental effort ratings. Zakeri et al. observed cardiac and ocular variables of 31 novices during simulated laparoscopic surgery. They showed that task difficulty can be predicted from the physiological measures^48^. Task difficulty could also be identified from eye movement events of surgical residents in endo-neurosurgery simulation^49^. Pupil metrics, blink rate, and gaze measures have also been generally reported as valid indicators of workload in surgery^46^. While physiological measures have been used to assess cognitive load during various medical procedures, no work has investigated this for vitreoretinal surgery.

**Figure 1:**
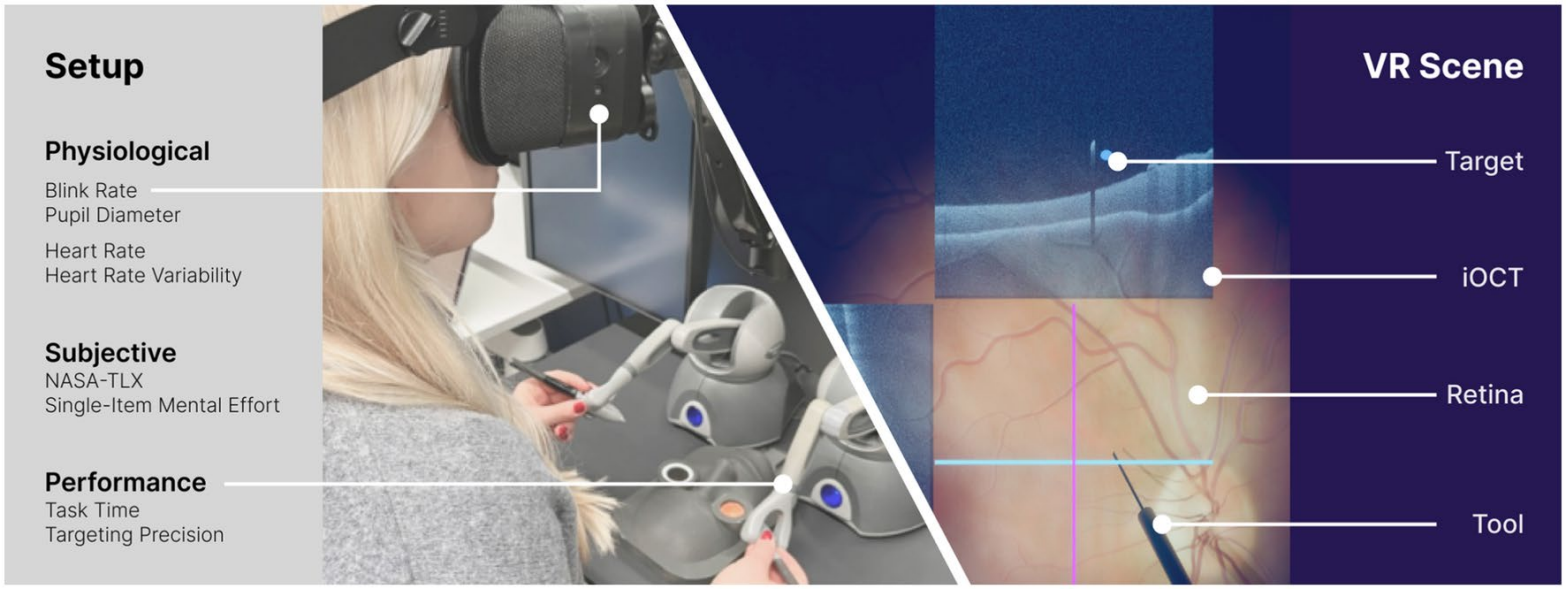
Left: Cognitive load measurements captured in the experiment setup. The eye surgery simulator consists of a VR head-mounted display and 3D haptic devices to steer the virtual tools in the surgery simulation; Right: VR scene (Mode 2 - iOCT) simulating the surgical scenario.

## Methods

For our user study, we leveraged an existing surgical simulation system (SynthesEyes GmbH, Germany), providing a virtual surgical scene setup for vitreoretinal interventions. Renderings of the surgery simulation were displayed to the users on a VR HMD, mimicking the view through the operating microscope. Two haptic 3D input devices (Geomagic Touch, 3D Systems GmbH, Germany) were used to steer the virtual surgical instruments inside the simulation scene (Figure 1). Motion scaling of 10 : 1 was applied to mitigate the effect of manual tremors and the different skill levels of individual novice users. However, no haptic feedback was provided. It is important to note that due to the delicate nature of retinal tissue, the majority of forces during retinal surgery are below the threshold of what can be perceived by human touch^50^. Surgeons are forced to rely on visual feedback in the absence of haptic feedback^51^. Thus, for our specific intraocular microsurgery task, visual feedback is sufficient to simulate the real surgical scene.

### Virtual reality visualizations

**Figure 2:**
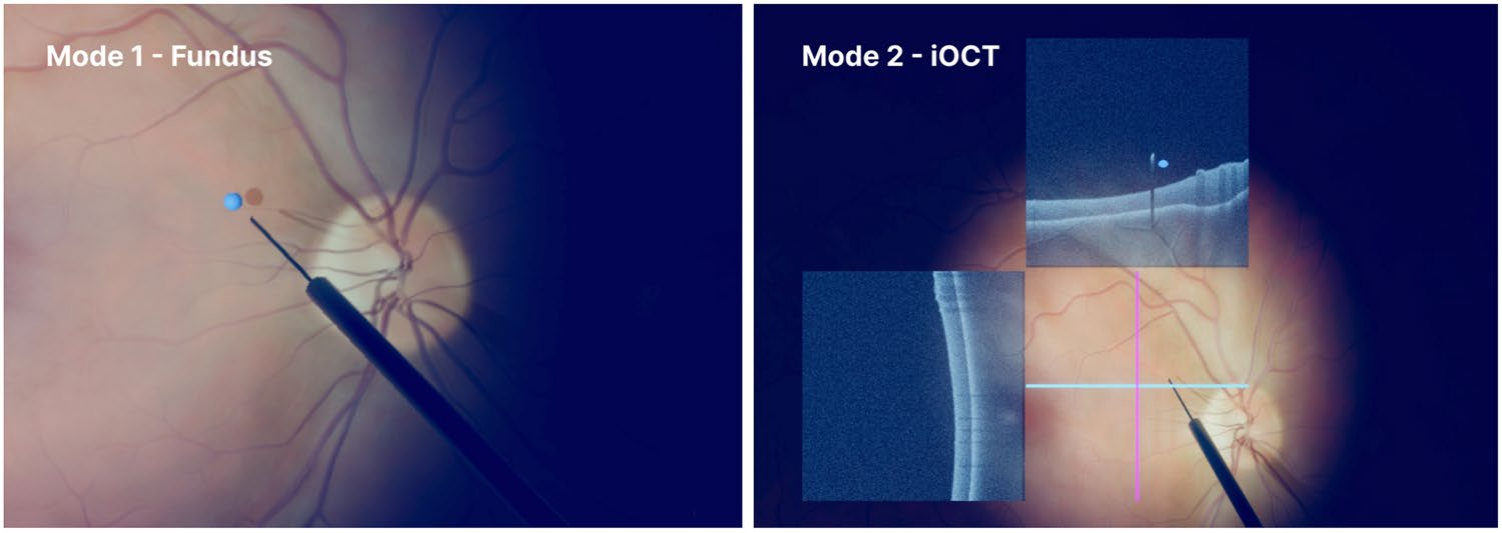
The two realistic vitreoretinal surgery scenarios compared in the user study regarding cognitive load during a targeting task on a virtual reality eye surgery simulator. Both simulated scenarios show a view of the retina, the surgical instrument, and a blue target sphere. Left: conventional microscope view without iOCT; Right: microscope view including two iOCT B-scans.

During the study, participants were asked to perform targeting tasks within two different virtual scene setups. To allow novice users to participate in the study, tasks were selected that did not require specific medical knowledge. The selected tasks primarily aim to increase the manual dexterity of the users to precisely navigate microsurgical tools in an ophthalmic surgical environment. In particular, during each trial, users had to navigate the tip of the surgical instrument to a small blue opaque target sphere. Such training tasks help to build essential manual skills to perform targeted surgical maneuvers required, for instance, during retinal membrane peeling or subretinal injections. For the user study, the targets were randomly positioned in the scene, however, located close to the retinal surface, and marked in a distinctive blue color. Once the instrument tip reached the desired position, the target color changed from blue to green to notify the user about the correct positioning. Participants were instructed to keep the instrument tip within the target sphere for a specific amount of time until the sphere disappeared. In addition, they were told not to touch the retina as this can lead to irreversible damage to a patient’s vision in real surgery. A red color effect was overlayed atop the microscope view in the simulation scene whenever the instrument touched the retina to provide a warning signal to the user. After each trial, the participants were asked to navigate the instrument back to a predefined area visualized as a larger sphere with a distinct translucent green color to enforce a similar starting position and criteria for each trial and user. The task was performed in two different scenarios, both mimicking realistic surgical scenes. One scenario simulated a traditional setup featuring the microscopic view, while the other scenario simulated a modern surgical setup with additional iOCT imaging. The two scenarios are depicted in Figure 2. In the following, these two modes are described in detail.

**Mode 1 - Fundus** In this scenario, users were provided a virtual simulation of the conventional view through the operating microscope without iOCT imaging. The haptic devices were employed to control the surgical instrument and the illumination probe. Users were asked to navigate the surgical instrument toward the target. To enhance depth perception, they were instructed to use the illumination probe to generate the typical instrument shadow, as well as the shadow of the target sphere on the retina. Simultaneously aligning the tip of the instrument with the target and the tip of the instrument’s shadow with the target’s shadow provides the visual cues of correct positioning.

**Mode 2 - iOCT** The second virtual scenario integrates simulated iOCT B-scans into the microscopic view. The B-scans are visualized next to the imaging area as a semi-translucent overlay, as provided in current surgical systems. The instrument and the illumination probe were controlled as in Mode 1 - Fundus. While the target sphere was analogously positioned randomly above the retina, as opposed to the microscope-only scenario, it was only visualized in the iOCT B-scan cross-sections, forcing participants to not only use the microscopic image as in Mode 1. Instead, users were required to look at the B-scan overlay for target localization but at the same time to position the instrument tip at the spatially separated iOCT scanning area at the retina. This provides a realistic scenario in which a micrometer-thin anatomical structure is hardly visible in the microscopic view but can be visualized using iOCT imaging. In contrast to relying on the shadow information in Mode 1, in this scenario, the B-scan cross-sections were used to estimate the distance between the instrument and the target. Thus, users were required to process and integrate visual information from the spatially separated microscopic view and iOCT images, potentially increasing the cognitive demand on the users.

Summarizing the study setup, two realistic surgical scenarios with different visual complexity, potentially imposing different levels of cognitive load, were provided in a simulated environment. In both scenarios, the same targeting task was performed by the participants.

## User study

We conducted a user study to investigate whether iOCT results in increased cognitive load in novice users during vitreoretinal surgery tasks. The study tested the following hypotheses:

- H1: iOCT-guided surgery (Mode 2 - iOCT) will result in increased cognitive load compared to microscope-guided surgery (Mode 1 - Fundus).
- H2: Physiological measures of the eyes (BR, PD) and heart (HR, HRV) are indicative of subjective cognitive load (NASA-TLX, self-reported mental effort) during simulated eye surgery tasks.
- H3: iOCT improves surgical precision by enhancing the depth-related targeting capabilities of users during simulated eye surgery.

### Ethics statement

Ethical approval for this study was obtained from the Ethics Committee of the Technical University of Munich. The research was performed in accordance with relevant guidelines and regulations. Informed consent was obtained from all participants prior to the start of the study.

### Participants

Seventeen volunteers with a mean age of 30 ± 4.29 years participated in the study. Six indicated to be women and eleven to be men. The study included participants with medical and non-medical backgrounds. Their occupations ranged from engineer to pilot and physician. None of the participants indicated to suffer from a vision impairment. Regarding the participants’ experience with eye surgery tasks, twelve indicated to have never performed such tasks before, four stated to have performed eye surgery tasks on a simulator before and one participant was highly familiar with the tasks and had previously assisted in eye surgery.

### Experimental variables

We selected proven physiological measures of cognitive load that could be easily obtained in a surgical training setup. Many VR HMDs already come with integrated eye tracking, making ocular measurements the most accessible physiological measures of cognitive load in VR-based surgery training. Additionally, heart rate and heart rate variability are simple to capture by placing three chest electrodes, requiring less setup time than, for example, electroencephalography-based brain activity measurements. This setup efficiency makes ocular and cardiac measures more suitable for surgical training, as setup time should be minimized to accommodate the typically busy schedules of clinicians.

### Study procedure

**Figure 3:**
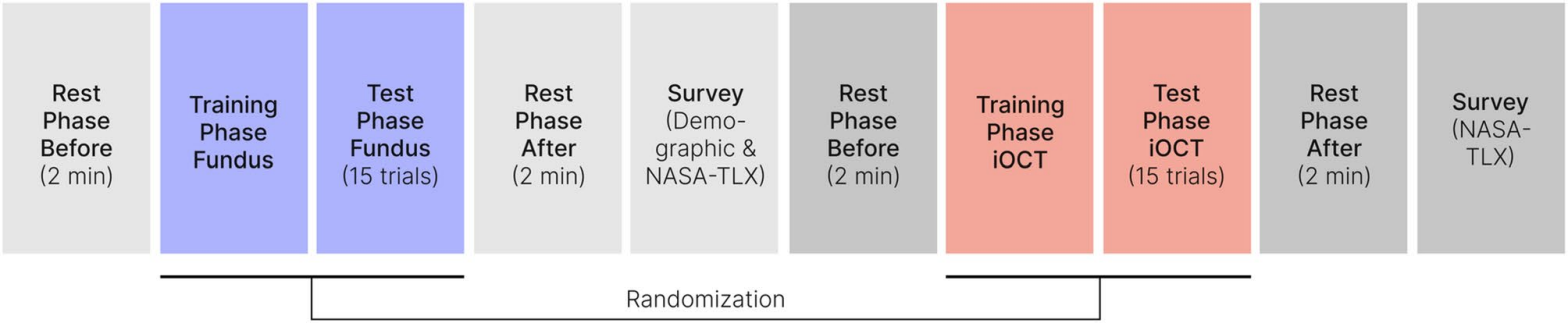
Experiment protocol of the user study.

While HR is easy to measure, it is also easily influenced by physical or psychological factors. To control for confounding effects on HR measurements during our study, a controlled experiment in a calm and neutral environment was established to minimize external emotional influences. Sufficient rest periods after the participant’s arrival and before the experiment’s start were ensured to reduce any pre-existing physical stress or emotional arousal (Figure 3). We further controlled for caffeine intake and collected baseline measurements to account for individual differences between participants.

Prior to the study the participants received an introduction to eye anatomy and the surgical task they would be performing on the simulator. In a consecutive step, we attached the electrodes of a three-channel ECG to their chest to measure their HR and HRV during the study. Participants were then asked to take a seat at the surgery simulator. We first performed an eye calibration in the VR HMD, followed by the first rest phase, which showed a two-minute nature video. This allowed us to capture the participants’ individual resting heart and eye measures. Nature videos were chosen as they have been proven to promote relaxation and physiological restoration^52^. After the rest phase, the participants entered the training phase. During training, they were able to familiarize themselves with the targeting task using the simulator without any time pressure. Once they understood the basic functionality of the system and the task goal, they started the actual testing phase using one of the two study modes. They had to perform 15 trials which linearly increased in difficulty over time. The time the users had to hold the instrument tip on the target location was linearly increased (from 2 to 4 seconds) throughout the trials. The increase in difficulty was chosen to impose added demand on the test subjects. The 15 targets were placed in various locations above the retina (Mode 1) or on the iOCT image (Mode 2). The order of the targets was randomized. At the end of every third trial, participants were asked to self-report a single-item measure of mental effort on a scale from 0 to 20. Following the test phase, they again entered a two-minute rest phase. Once the second rest phase was completed, the participants exited the VR HMD to fill out an online questionnaire on a dedicated computer. The questionnaire included some demographic questions, a raw NASA-TLX, followed by three qualitative questions regarding their experience with the system. This process of eye calibration, rest, train, test, and rest phase was repeated a second time for the second study mode. The order in which the participants were exposed to the two modes was randomized.

### Data collection and analysis

To facilitate data analysis, participant data needed to be either time-warped or aggregated to allow synchronization. Since in this study time-specific measurements were less important, we simplified the problem to two groups of 15 trials per user, thus each group corresponds to one study mode. In each of the trials, which took from 10 to 384 seconds, the measurements were averaged among all the samples. In the absence of trials in the rest phases, a similar approach was taken, whereby the data for the entire phase was aggregated using the average value. To establish alignment of the self-reported mental effort rating and the physiological data grouped by *trial, visualization mode, user*, the mental effort values were linearly interpolated for the trials in between the reporting, as the self-reporting happened every third trial.

#### Ocular measurements analysis

We used the Varjo Aero integrated eye tracking to collect the ocular measures in Unity. Data were sampled at 80 Hz. All data processing and analysis were performed using Python. Among the provided eye tracking measurements, we focused on the binocularly recorded pupil diameter and eye openness. We calculated the blink rate as the number of changes in the eye openness variable per minute divided by two. For calculating the pupil diameter we performed preprocessing by removing missing or invalid pupil data caused by blinks or tracking inaccuracy. We further calculated the mean for short, 200 ms sections within a trial to produce a time series. Since binocular data were available, a third mean pupil size time series was generated. In a next step, subtractive baseline correction was performed to increase statistical power^53^. The average pupil diameter in millimeters during the two-minute rest phase before the task was computed and subtracted from the pupil diameter values during the task phase. The same correction was applied to the BR.

#### Cardiac measurements analysis

The cardiac measures were acquired using the BiosignalsPlux (PLUX Wireless Biosignals S.A., Portugal) wireless ECG. Samples were collected via the Opensignals software at 1000Hz. Based on the system timestamps the heart data was fused with the eye tracking data. Heart rate and heart rate variability were computed from the raw ECG data using HeartPy^54^. The provided beats per minute (BPM) value was used for HR. We used the root mean square of successive RR interval differences (RMSSD) as a measure of HRV as it is one of the HRV measurements that can be applied to ultra-short-term recordings of less than five minutes^30^. Researchers have shown the validity of RMSSD for the analysis of mental stress for up to time periods as short as 10 seconds^55^. We employed 30-second time intervals, utilizing process_segmentwise function from the HeartPy library for this purpose.

**Figure 4:**
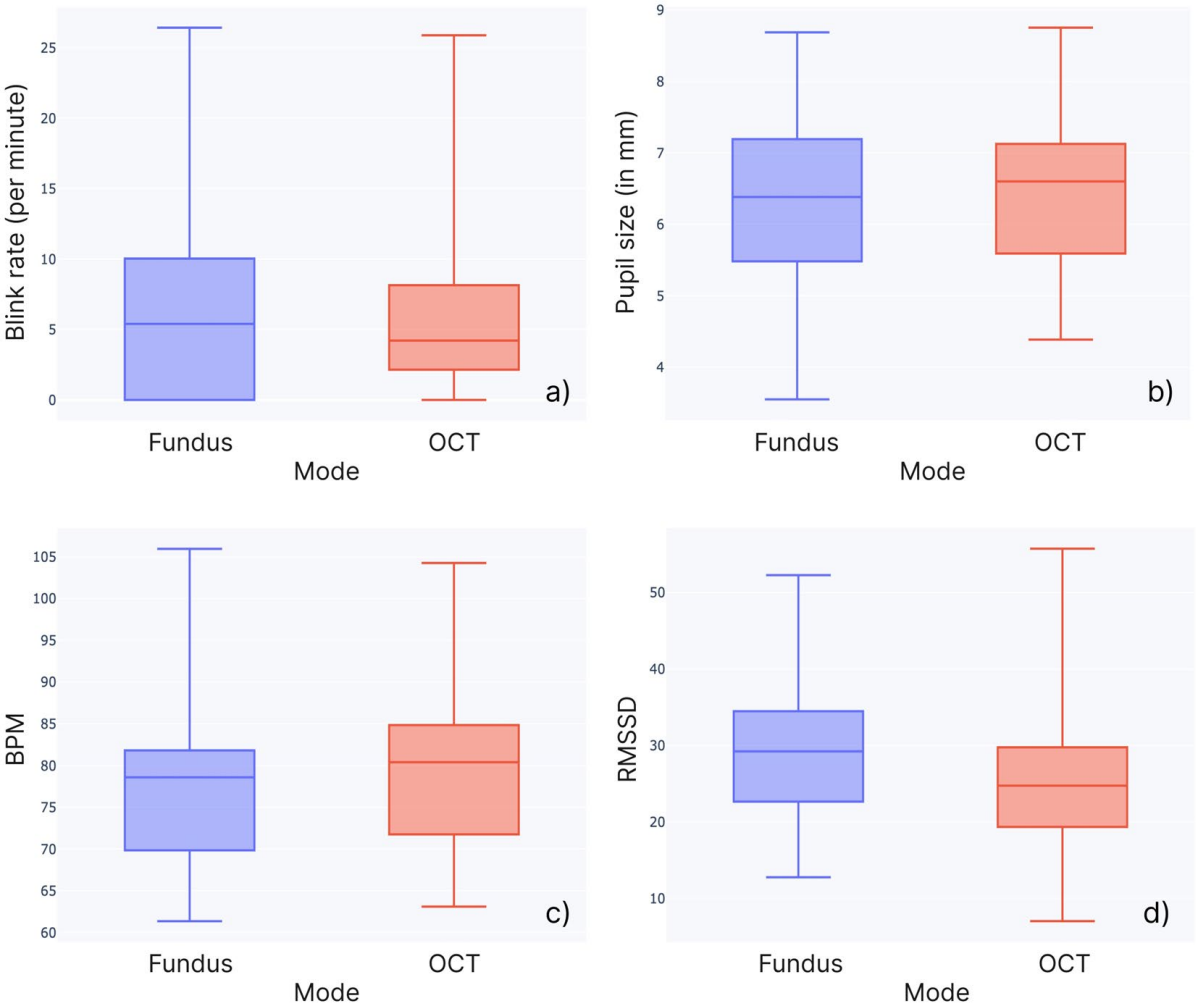
Boxplots of baseline corrected (**a**) blink rate (blinks per minute), (**b**) pupil diameter (in millimeters), (**c**) heart rate (in beats per minute), (**d**) heart rate variability (RMSSD) during task for both modes, Fundus (blue) and iOCT (red).

## Results

**Table 1:** Means, standard deviations (std), and p-values of the physiological measures of cognitive load (blink rate (BR), pupil diameter (PD), heart rate (HR), heart rate variability (HRV)) for the Fundus and iOCT modes for the different study phases (rest before task, task, rest after task). For strictly positive values, like the measurements here, it is very unlikely to have a mean of the distribution less than the $\text {std}$. Nonetheless, such an occurrence could potentially be attributed to noise present in the data.

		Rest Before		Task		Rest After		Rest Before / Task	Rest After / Task
		mean	std	mean	std	mean	std	p−value	p−value
Fundus	BR (blinks per minute)	24.14	15.70	8.06	6.53	42.11	27.27	**<0.01**	**<0.001**
	PD (mm)	5.00	0.84	6.30	1.10	5.67	1.04	**<0.001**	**<0.05**
	HR (beats per minute)	75.55	8.39	77.81	9.63	78.40	9.68	>0.05	>0.05
	HRV (rmssd)	28.77	8.95	29.68	7.71	27.81	7.71	>0.05	>0.05
iOCT	BR (blinks per minute)	29.52	20.10	*8.60*	*10.04*	38.86	20.54	**<0.001**	**<0.001**
	PD (mm)	5.37	0.87	6.48	1.09	4.78	0.88	**<0.01**	**<0.01**
	HR (beats per minute)	75.82	10.98	79.25	11.07	77.33	11.31	>0.05	>0.05
	HRV (rmssd)	30.63	12.03	26.35	8.95	29.06	7.57	>0.05	>0.05

### Physiological cognitive load

We analyzed the physiological data for differences between the microscope-guided (Mode 1 - Fundus) and iOCT-guided (Mode 2 - iOCT) scenarios, as well as the rest and task phases. A Shapiro-Wilk test showed a normal distribution of the physiological data. The interquartile range method was used for outlier removal. When sample lengths differed after outlier removal, values were randomly sampled from the larger sample to match the size of the smaller sample. Since we used a within-subject study design, paired samples t-tests were used to test for significance.

The tests showed that both ocular measures (blink rate and pupil diameter) were significantly different between the rest phase before and the task, as well as between the task and the rest phase after (Table 1). The paired samples t-tests further showed significant differences in HR (Figure 4c) and HRV (Figure 4d) between the Fundus and iOCT mode. No significant differences in BR (Figure 4a) or PD (Figure 4b) were seen between modes (Table 2). The results further show a significant drop in blink rate (Fundus: ($p<0.01$); iOCT: $p<0.001$) and a significant increase in pupil diameter (Fundus: $p<0.001$; iOCT: $p<0.01$) during task activity for both modes. For the cardiac measures of HR and HRV, no significant differences between rest and task phases were found.

### Performance

A Shapiro-Wilk test showed a normal distribution of the task time data. The interquartile range method was used for outlier removal. A paired samples t-test showed a significant difference in task time between the microscope-guided (Fundus) and iOCT-guided surgery (Table 2). Users performed the task significantly faster ($p<0.001$) using Mode 1 - Fundus.

**Figure 5:**
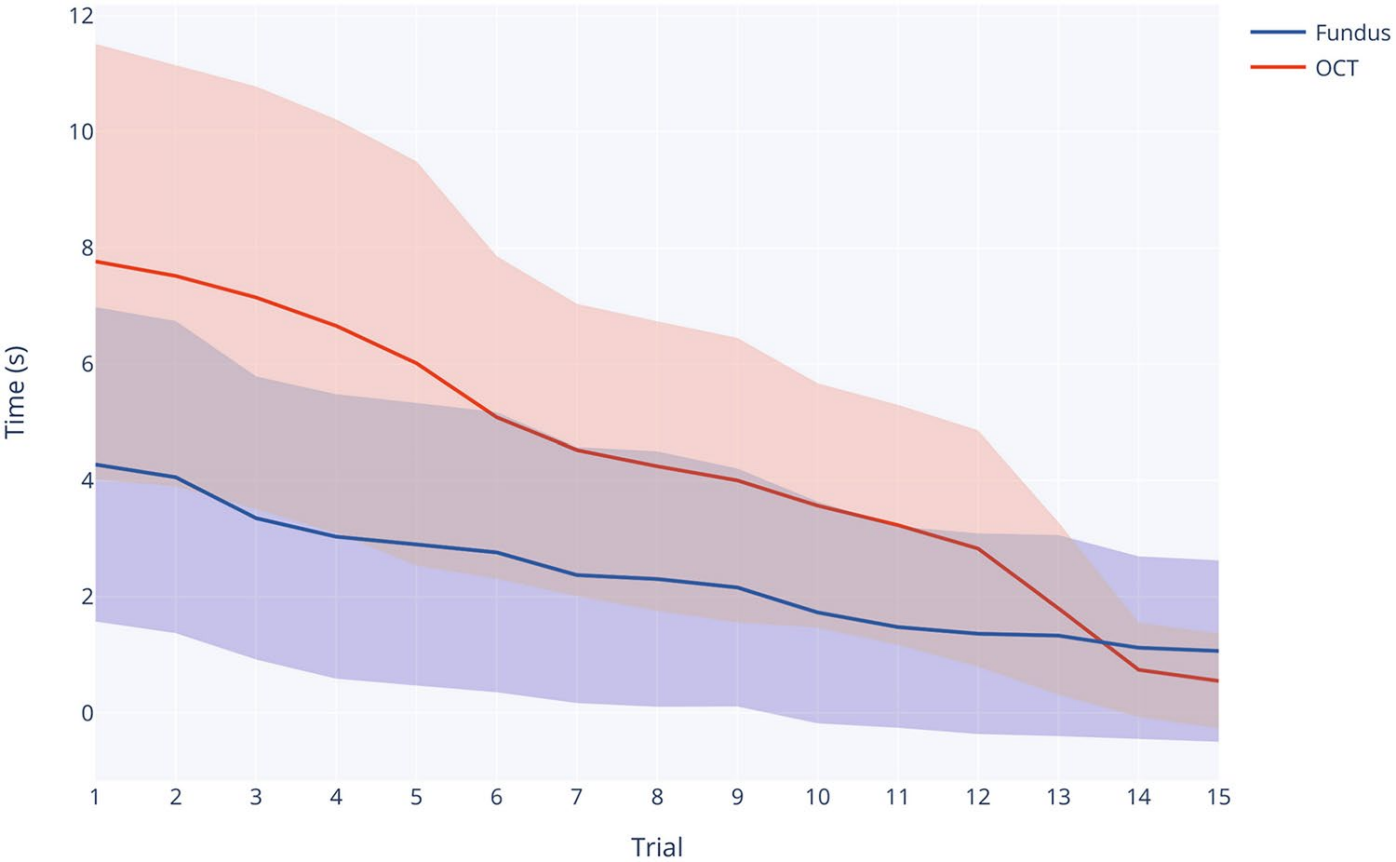
Depth-related targeting precision across trials. The *y*-axis indicates the total time the tool tip remained below the target during the task.

We further looked at depth-related targeting precision as a measure of surgical skill in retinal procedures. It is crucial for the surgeon to maintain awareness of the retinal topography to avoid causing damage to the retinal layers. Ideally, during the procedure, the tool should not be positioned below the target, to minimize the risk of injury. In Figure 5, the total time each user spent with the tooltip below the target while approaching the target is depicted. Although users improved their targeting performance in both modes as they progressed through the trials, it can be observed that their initial performance in iOCT mode was poor. However, with learning and adaptation, performance in the iOCT mode improved at a faster rate compared to the Fundus mode, eventually surpassing it. Furthermore, user performance variance in iOCT mode also converged over time, whereas the variance remained relatively constant in Fundus mode.

**Figure 6:**
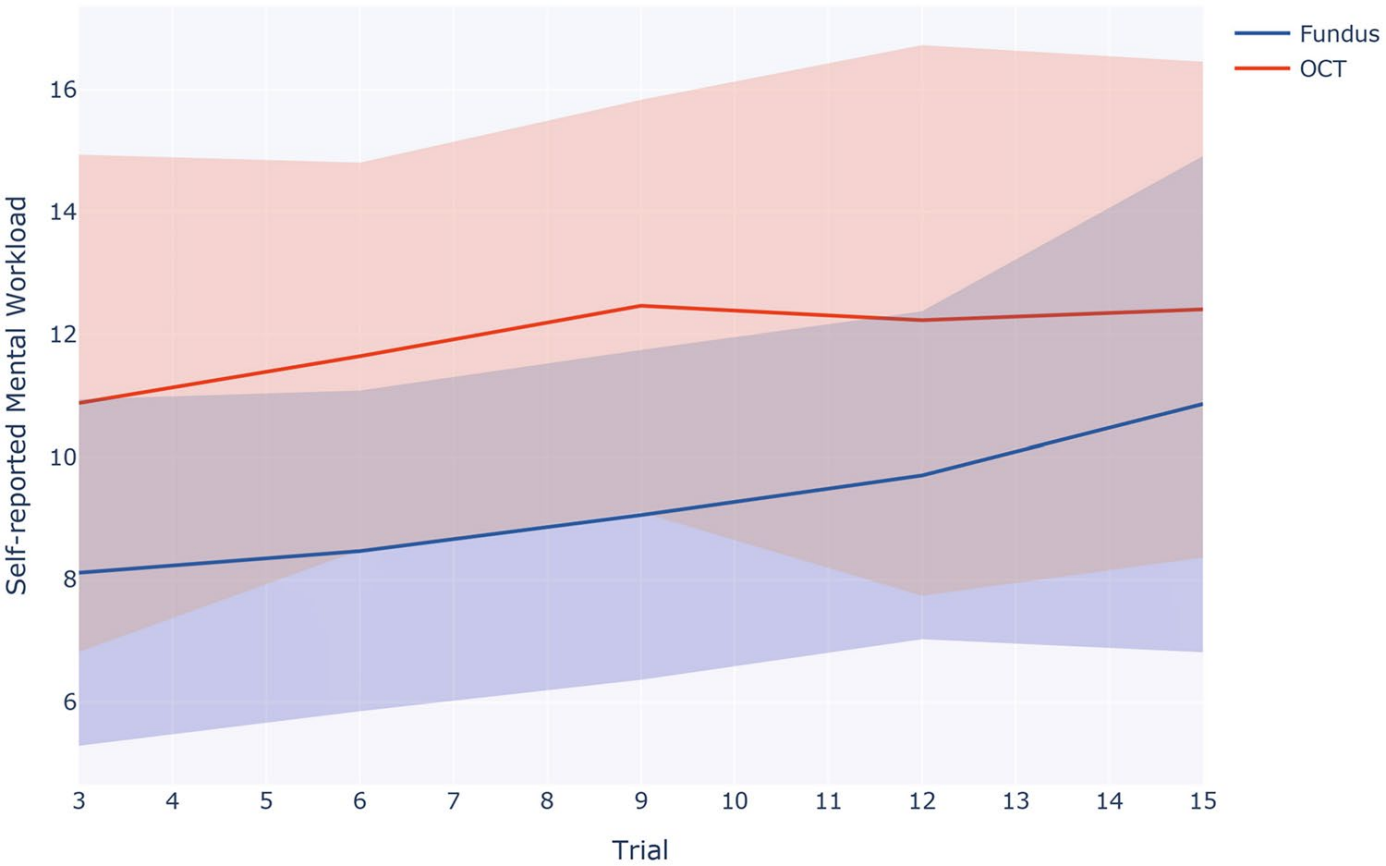
Self-reported mental effort ([0-20] lower better) across trials for Fundus (blue) and iOCT mode (red).

**Table 2:** Means, standard deviations (std) and p-values of the performance and physiological cognitive load measures before and after baseline correction (corr): blink rate, pupil diameter, heart rate, heart rate variability, task time per trial.

Physiological & Performance	Fundus	iOCT	Fundus/iOCT
	mean ± std	mean ± std	p-value
Blink rate (blinks per min)	8.06 ± 6.53	8.60 ± 10.04	>0.05
Pupil diameter (mm)	6.30 ± 1.10	6.48 ± 01.09	>0.05
Heart rate (bpm)	77.81 ± 9.63	79.25 ± 11.07	**<0.01**
Heart rate variability (rmssd)	29.68 ± 7.71	26.35 ± 8.95	**<0.05**
Blink rate corr (blinks per min)	-13.37 ± 19.78	-18.25 ± 21.90	>0.05
Pupil diameter corr (in mm)	1.14 ± 0.81	0.92 ± 0.65	>0.05
Heart rate corr (bpm)	1.54 ± 3.65	3.85 ± 3.64	>0.05
Task time (s)	28.72 ± 25.15	49.60 ± 51.32	**<0.001**

**Table 3:** Means, standard deviations (std) and p-values of the subjective measures: NASA-TLX ([0-100], lower better, except Performance), self-reported mental effort ([0-20] lower better), distance perception effort ([1-5] lower better).

Subjective	Fundus	iOCT	Fundus/iOCT
	mean ± std	mean ± std	p-value
TLX Overall	47.23 ± 11.96	62.14 ± 10.58	**<0.001**
TLX Mental	56.25 ± 22.47	76.25 ± 13.10	**<0.001**
TLX Physical	51.25 ± 23.90	61.25 ± 25.26	**<0.05**
TLX Temporal	35.62 ± 18.60	51.87 ± 20.72	**<0.05**
TLX Performance	46.87 ± 21.20	43.12 ± 17.01	>0.05
TLX Effort	55.62 ± 19.98	76.25 ± 15.43	**<0.001**
TLX Frustration	42.50 ± 20.16	63.12 ± 22.12	**<0.01**
Mental effort	9.31 ± 2.92	12.17 ± 3.27	**<0.001**
Distance perception effort	3.50 ± 1.12	2.56 ± 0.79	**<0.01**

### Subjective cognitive load

Besides the impact of iOCT on physiological and performance measures, we also evaluated its influence on subjective cognitive load. A Shapiro-Wilk test showed the normality of the raw NASA-TLX and single-item mental effort data. The interquartile range method was used for outlier removal. Paired samples t-tests showed a significant effect of iOCT on both subjective ratings.

**Figure 7:**
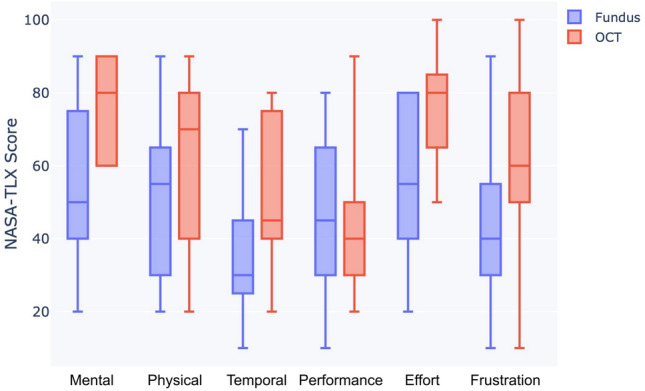
Boxplot of NASA-TLX scores ([0-100], lower better, except Performance - higher better) for all six subscales (Mental Demand, Physical Demand, Temporal Demand, Performance, Effort, Frustration) for Fundus (blue) and iOCT (red).

The NASA-TLX subscales of Mental Demand ($p<0.001$), Physical Demand ($p<0.05$), Temporal Demand ($p<0.05$), Effort ($p<0.001$), and Frustration ($p<0.01$), as well as the overall average NASA-TLX ($p<0.001$) across all scales, showed a significantly higher task load when using the iOCT mode (Table 3). No significant difference was found for the Performance subscale. A boxplot comparing the NASA-TLX scores of the two modes can be found in Figure 7.

The single-item mental effort question asked every third trial throughout the study indicated a significantly higher ($p<0.001$) average mental effort when using Mode 2 - iOCT (Table 3). We visualized the evolution of the self-reported mental effort over the course of the study in Figure 6. An increase in mental effort throughout the simulated surgery is shown for both modes, however, mental effort always remained greater during iOCT-guided surgery.

Conversely, the self-reported distance perception effort was significantly lower ($p<0.01$) in iOCT than in Fundus mode (Table 3).

### Qualitative feedback

When users were asked to provide qualitative feedback after the study, a common remark was that iOCT mode led to higher cognitive demand due to simultaneously controlling light and instrument while focusing on the iOCT images. When users were asked about ways to improve the iOCT visualization, suggestions included only displaying the relevant OCT cross-section containing the target. This comment could be related to the visual overload that users experienced when provided with the maximum amount of imaging data available. To improve the surgical interface, participants suggested the addition of cues regarding the distance between the instrument and the retina and an auditory signal to indicate when the instrument is in close proximity to the retina.

Expert feedback from a qualitative interview with a senior vitreoretinal surgeon suggests that iOCT helps to improve surgery technique as it serves as a constant feedback mechanism during vitreoretinal surgery, enabling surgeons to evaluate the outcome of their actions right away. This real-time feedback leads to faster skill acquisition and technique refinement. Therefore, it is important to not only use iOCT in surgery, but to already incorporate it into training systems to enable residents to benefit from this performance feedback during learning.

## Discussion

This work evaluated the effect of intraoperative optical coherence tomography on cognitive load during vitreoretinal surgery training. The user study confirmed our hypothesis that iOCT-guided surgery results in increased cognitive load among novice users. This was shown by the physiological measures of the heart, the performance metrics, and the subjective cognitive load ratings. Heart rate and heart rate variability proved significantly different between the two eye surgery modes. The iOCT mode resulted in higher average HR $p<0.01$, associated with increased stress^34^, and lower average HRV $p<0.05$, indicating greater mental workload^56^.

To contextualize these findings, it is important to consider the different demands associated with iOCT versus microscopic view only. Unlike the microscopic view in the Fundus condition, the iOCT condition required the user to interpret additional depth cues, process multi-layered image data, and integrate this added visual information into their surgical actions. This requires the user to switch focus between multiple information sources, thus contributing to increased cognitive strain and physiological responses such as elevated HR and reduced HRV. Furthermore, interacting with novel imaging modalities, such as iOCT, which provides cross-sectional views of the retina, could further explain the increased cognitive load. These aspects influence the complexity of iOCT-guided vitreoretinal surgery and call for further investigation. Understanding how these factors individually contribute to cognitive load would help to inform a user interface design that enhances the training experience.

Based on previous works, we expected the presence of iOCT and its associated increase in visual complexity to result in a larger average pupil diameter and lower average blink rate^36,40^. However, no significant differences could be detected for the ocular measures. Inter-phase changes in luminance might have impacted the reliability of the eye measurements. The videos during the rest phase were of higher average grayscale intensity measured in pixel brightness (range: 0 - 255) than the two modes. However, we controlled for similar average pixel brightness within the rest (102.90 - 115.06) and task phase (Fundus - 53.06, iOCT - 57.56), resulting in a consistent effect across the two conditions. Ocular responses did, however, show significant changes between rest and task phases for both visualizations. This is in line with the findings in a previous study that reports high sensitivity of physiological measures to task-rest differences^29^.

Although no significant differences in ocular measures were observed between the microscopic and iOCT-guided view, these metrics remain valuable indicators of cognitive load. In tasks with substantial cognitive load, such as targeting in vitreoretinal surgery, the effects of cognitive load on pupil response overshadow the effects of physiological arousal^35^. Thus, in our case, increased pupil diameter can be associated with increased task difficulty and mental demand^37^. Similarly, an increase in blink rate can reveal a heightened workload^39^. Future analysis could examine ocular responses in more detail, potentially exploring their relationship with engagement or fatigue during surgical training.

Except for the ocular metrics, we did see correlations between the physiological, performance, and subjective cognitive load measures. Heightened cognitive load during iOCT-guided surgery was, for example, equally reflected in the HR, HRV, task time, and mental effort rating. Therefore, our work showed that physiological responses of the heart can be used as a substitution or addition to conventional subjective cognitive load ratings such as the NASA-TLX to evaluate vitreoretinal surgery tasks.

Although iOCT-guided surgery resulted in a greater average cognitive load, it allowed users to perform the surgical task with increased depth-related targeting precision by the end of the study. The line plot in Figure 5 depicts a steeper learning curve of iOCT-guided surgery, ultimately leading to smaller variance. This finding aligns with the participants’ subjective rating. A 5-point Likert-scale question revealed that participants found it easier to perceive tool-retina distance during iOCT-guided surgery. The learnability of iOCT is also reflected in Figure 6 showing an initially higher but eventually plateauing mental effort during iOCT use. Both indicators imply that novice users initially require more practice to become proficient with iOCT guidance but ultimately adapt to the novel imaging modality. These findings align with cognitive load theory, which suggests that an initial increase in cognitive load is indicative of learners acquiring schemata relevant to long-term skill retention^20^. However, once a schema is established in long-term memory, working memory load is reduced^57^.

Future studies could extend this analysis to investigate whether experienced surgeons exhibit lower cognitive load when using iOCT compared to novices. A previous study comparing expert and novice performance for four vitreoretinal surgery tasks on a VR simulator showed that experts performed membrane peeling and core vitrectomy with enhanced precision and reduced task time^58^. It would be interesting to examine if the effect of expertise also pertains to cognitive load or whether additional attentional resources are bound by iOCT regardless of training.

The improved surgical precision and depth perception suggest that iOCT images are a helpful addition to vitreoretinal tasks. To maximize their usability and reduce the cognitive strain they impose, the interface has to be redesigned with attention to information presentation. Reducing redundant visual cues or implementing adaptive overlays to blend iOCT and the microscopic view could enhance the usability of iOCT. Another consideration is the impact of imaging artifacts on cognitive load. Instrument-induced shadows and mirroring effects could introduce additional cognitive demands by requiring users to interpret ambiguous or inconsistent iOCT images. While our study did not quantify the prevalence of these imaging artifacts, future work could examine how these aspects affect cognitive load and task performance in iOCT-guided vitreoretinal surgery.

Another avenue of future work could be a focus on cognitive load adaptive user interfaces (UIs) for surgery and surgery training. This will become more relevant with the increase of novel sensing technologies such as iOCT, hyperspectral imaging, or ultrasound. While they provide more and more anatomical and physiological information during surgery, this data richness pushes the limits of the human perceptual system. In advanced surgical settings with multidimensional information streams and complexities beyond human sensing, intelligent UIs need to take human perception and cognitive load into consideration when transmitting information to surgeons. There is a growing body of research that has experimented with adapting UIs based on cognitive load levels, for example, in Mixed Reality settings, using, e.g., ocular^59^, brain^60^ or skin response measures^61^. This could, for example, be applied to surgical training to create learning applications that are adapted to the user’s working memory capacity, resulting in learning experiences that improve the transfer of knowledge to long-term memory and overall enhance surgical education. Beyond training, adaptive UIs aware of the surgical task could be integrated into surgery, managing the user’s cognitive load and improving surgeon performance and patient outcome.

## Conclusion

We presented the first study to evaluate cognitive load during iOCT-guided vitreoretinal surgery. Our results show a steep learning curve for iOCT-guided tasks and improved surgical precision. However, despite the substantial performance benefits iOCT provides in vitreoretinal procedures, our study suggests that this comes at the cost of increased cognitive load. It is, therefore, essential to keep developing advanced means of presenting iOCT data, such as advanced visualization or sonification techniques that enhance its effectiveness and improve user comprehension. Incorporating cognitive load evaluations into the design and development of new surgical systems will be beneficial to ensure that advanced imaging technologies such as iOCT are both intuitive and clinically effective. We envision this paper as the beginning of a series of studies that not only assess the cognitive load of novel technologies in surgery but also leverage these findings to improve the integration of advanced sensing through cognitive-load-informed interaction design.

## Data Availability

The datasets generated and analyzed during the current study are available from the corresponding author upon reasonable request.
